# Systemic Toxicity Profile of Chemotherapeutic Agents in a Wistar Rat Model: A Preliminary Report

**DOI:** 10.1155/jt/8811238

**Published:** 2025-11-19

**Authors:** Joseph Kathare, James Mbaria, Catherine Kaluwa, Issac Mapenay, Gervason Moriasi

**Affiliations:** ^1^Department of Public Health, Pharmacology, and Toxicology, University of Nairobi, P.O. Box 29053 00625, Nairobi, Kenya; ^2^Department of Medical Biochemistry, Medical School, Mount Kenya University, P.O. Box 342-0100, Thika, Kenya; ^3^Department of Biochemistry, Microbiology, and Biotechnology, School of Pure and Applied Sciences, Kenyatta University, P.O. Box 43844-00100, GPO, Nairobi, Kenya

**Keywords:** biochemical markers, chemotherapy-induced toxicity, electrolyte imbalances, hematological parameters, OECD guideline 425

## Abstract

Chemotherapeutic agents are pivotal in cancer management, yet their utility is constrained by pronounced systemic and organ-specific toxicity, necessitating rigorous preclinical evaluation to enhance their safe application in oncology. This study examined the hematological, biochemical, electrolyte, and histopathological effects of seven chemotherapeutic drugs—docetaxel (10 mg/kg body weight), doxorubicin (10 mg/kg), paclitaxel (4 mg/kg), oxaliplatin (4 mg/kg), cisplatin (2 mg/kg), cytarabine (50 mg/kg), and cyclophosphamide (10 mg/kg)—and a phosphate-buffered saline (PBS) control (10 mL/kg) in male Wistar rats (*n* = 40, eight groups of five), adhering to the Organization for Economic Co-operation and Development (OECD) Guideline 425. Doses were selected based on established protocols from prior studies. Docetaxel induced leukocytosis and thrombocytosis, whereas doxorubicin and cisplatin elicited leukopenia and lymphopenia. Biochemical profiling revealed hepatotoxicity (elevated aminotransferase, alkaline phosphatase, and bilirubin) in rats treated with paclitaxel and cytarabine and nephrotoxicity (increased urea and creatinine) in those receiving paclitaxel and cyclophosphamide. Electrolyte analysis indicated hyperkaliemia with oxaliplatin, contrasted by hypokalemia with doxorubicin and cisplatin. Histopathological assessment of liver, kidney, spleen, and stomach tissues, stained with hematoxylin and eosin (H and E), revealed subacute, organ-specific alterations in rats administered cytarabine (50 mg/kg), cisplatin (2 mg/kg), doxorubicin (10 mg/kg), and cyclophosphamide (10 mg/kg) compared to the control (10 mL/kg), especially liver vacuolation and sinusoidal narrowing, kidney tubular flattening and glomerular compaction, spleen white pulp hypocellularity with red pulp congestion, and stomach submucosal inflammation with glandular hyperplasia. Notably, no necrosis or fibrosis was observed, suggesting potentially reversible effects. These findings highlight the heterogeneous toxicity profiles of chemotherapeutic agents, advocating for personalized monitoring and protective strategies in clinical practice to optimize therapeutic safety and efficacy.

## 1. Introduction

Cancer exacts a severe toll worldwide, with the International Agency for Research on Cancer (IARC) estimating 19.3 million new cases and 10 million deaths globally in 2020, a burden projected to rise to 28.4 million cases by 2040 [1]. In Africa, there is a rapidly growing cancer incidence crisis, with 1.1 million new cases and 711,429 deaths reported in 2020, where sub-Saharan Africa (SSA) alone accounted for 801,392 cases and 520,158 deaths, driven by breast, cervical, and prostate cancers, with mortality expected to double by 2040 if no effective intervention is implemented [2]. In Kenya, cancer ranks as the third leading cause of death, with 44,726 new cases and 29,317 deaths in 2022, exacerbated by limited screening and oncology resources [1, 3].

Chemotherapy, a mainstay in the management of numerous malignancies, targets rapidly proliferating cancer cells with notable efficacy [4]. Chemotherapeutic agents exert their anticancer effects through various mechanisms including DNA intercalation, microtubule disruption, and inhibition of nucleic acid synthesis [5]. However, its therapeutic benefits are frequently offset by systemic toxicity, which compromises patient outcomes and constrains treatment success [6, 7]. Histopathological examination reveals characteristic patterns of cellular damage, such as nuclear pyknosis, karyorrhexis, and apoptotic bodies in malignant tissues following treatment. However, these cytotoxic effects lack selectivity, resulting in significant histomorphological alterations to healthy tissues [8, 9].

The deleterious effects of chemotherapeutic agents extend beyond neoplastic tissues to impact healthy cells, especially those with elevated mitotic activity, such as bone marrow, gastrointestinal epithelium, and renal tubules [10, 11]. Elucidating the organ-specific toxicity profiles of these agents is imperative for devising strategies to ameliorate their adverse effects and enhance the safety of chemotherapy regimens [12]. Besides, the hematopoietic system demonstrates histopathological vulnerability, with chemotherapy-induced marrow hypoplasia showing reduced cellularity, fatty replacement, and maturation arrest across all lineages. These changes correlate clinically with leucopoenia, anemia, and thrombocytopenia [13]. Despite therapeutic efficacy, comprehensive histopathological correlates of chemotherapy toxicity remain incompletely characterized, particularly in sub-Saharan populations where postmortem and biopsy studies are limited. This study investigated the systemic impact of diverse chemotherapeutic agents, with a particular focus on their influence on hematological, biochemical, and electrolyte parameters, and histopathological characteristics in a rodent model. Integration of histopathological assessment with clinical monitoring could enhance toxicity prediction and guide personalized treatment regimens to improve patient outcomes.

This study delineates the distinct toxicological profiles of seven commonly used chemotherapeutic agents within a standardized Wistar rat model, thereby advancing understanding of chemotherapy-induced systemic toxicity and addressing a notable gap in the literature, where most preclinical studies examine individual drugs or isolated organ systems [14–16]. By systematically evaluating hematological, biochemical, electrolyte, and histopathological alterations under identical conditions, the research provides a holistic and directly comparable toxicity profile that holds translational value for clinicians selecting regimens, particularly in resource-limited settings where toxicity monitoring may be constrained. The findings underscore the necessity of routine monitoring of hematological, biochemical, and electrolyte parameters in chemotherapy recipients, while also highlighting the potential to develop tailored therapeutic strategies that balance efficacy with safety and guide personalized monitoring protocols aligned with drug-specific risk patterns [17, 18]. Although the precise molecular mechanisms underlying the observed toxicities were not determined, the work offers a strong evidence base for mitigating chemotherapy-related risks, with the ultimate goal of enhancing patient safety, quality of life, and clinical outcomes.

## 2. Materials and Methods

### 2.1. Experimental Design

This study was designed to evaluate the systemic effects of eight chemotherapeutic agents on hematological, biochemical, and electrolyte parameters in a rodent model, in accordance with the Organization for Economic Co-operation and Development (OECD) Guideline 425 using the up-and-down procedure [19]. The agents tested included docetaxel, doxorubicin, paclitaxel, oxaliplatin, cisplatin, cytarabine, cyclophosphamide, and a phosphate-buffered saline (PBS) control. A total of 40 adult male Wistar rats, aged 8–10 weeks and weighing 190–220 g, were used for the study. The animals were randomly divided into eight groups (*n* = 5 per group), with each group receiving a single chemotherapeutic agent or PBS control via intraperitoneal injection. The doses administered were based on established protocols from previous studies and were as follows: docetaxel (10 mg/kg body weight), doxorubicin (10 mg/kg), paclitaxel (4 mg/kg), oxaliplatin (4 mg/kg), cisplatin (2 mg/kg), cytarabine (50 mg/kg), cyclophosphamide (10 mg/kg), and PBS (10 mL/kg).

### 2.2. Experimental Animals and Ethical Considerations

Wistar rats aged between 8 and 9 weeks were obtained from the Animal Breeding Unit of the Department of Public Health, Pharmacology and Toxicology at the University of Nairobi. The animals were housed under standard laboratory conditions and were maintained on a 12-h light/12-h dark cycle. They were kept in well-ventilated cages, with softwood shavings used as the bedding material. All rats were provided with standard rodent pellets and clean water *ad libitum*. Prior to the commencement of the experiments, the animals were acclimatized to laboratory conditions for 72 h. Throughout the study, they were handled humanely, and all procedures involving their care and use were conducted in strict accordance with the guidelines established by the National Research Council for the ethical treatment of laboratory animals [20]. Upon completion of the study, the animals were disposed of in accordance with the same ethical guidelines. This study received ethical approval from the Bioethics and Animal Use and Care Committee (BAUEC) of the University of Nairobi (REF: FVM BAUEC/2023/430). Subsequently, a research permit was granted by the National Commission for Science, Technology and Innovation (NACOSTI), under License No: NACOSTI/P/24/35,134.

### 2.3. Administration of Chemotherapeutic Agents

The chemotherapeutic agents were prepared according to manufacturer's guidelines and diluted in sterile PBS to achieve the desired concentrations. Each agent was administered as a single intraperitoneal injection on Day 1 of the experiment, following OECD Guideline 425 for acute toxicity testing [19]. The PBS control group received an equivalent volume of sterile PBS. Following administration, the animals were monitored daily for 14 days for signs of toxicity, including changes in body weight, activity levels, and food and water intake. Clinical observations were recorded systematically to identify any adverse effects, as recommended by OECD [19]. Mortality, morbidity, and behavioral changes were documented to assess the acute toxic effects of the agents.

### 2.4. Blood Sample Collection and Processing

On Day 14 post-administration, the animals were anesthetized using ketamine (75 mg/kg) and xylazine (10 mg/kg) via intraperitoneal injection, in compliance with OECD Guideline 425 [19]. Blood samples were collected via cardiac puncture into EDTA-coated tubes for hematological analysis and plain tubes for biochemical and electrolyte analysis. The blood samples were centrifuged at 3000 rpm for 15 min to separate plasma and serum, which were stored at −80°C until analysis.

### 2.5. Hematological Analysis

Hematological parameters, including white blood cell (WBC) count, red blood cell (RBC) count, hemoglobin (Hb) concentration, platelet count, lymphocyte, neutrophil mean corpuscular volume (MCV), mean corpuscular hemoglobin (MCH), and hematocrit (HCT), were measured using an automated hematology analyzer (Mindray BC-5000).

### 2.6. Biochemical Analysis

Biochemical markers, including aspartate aminotransferase (AST), alkaline phosphatase (ALP), gamma-glutamyl transferase (GGT), albumin (ALB), total protein (TP), direct bilirubin, total bilirubin, urea, and creatinine, were quantified using commercially available assay kits (Manufacturer) and a semi-automated biochemistry analyzer (Mindray BS-230). All assays were performed in replicate to ensure accuracy and reproducibility.

### 2.7. Electrolyte Analysis

The concentrations of sodium (Na^+^), chloride (Cl^−^), and potassium (K^+^) in serum were measured using an ion-selective electrolyte analyzer (Mindray BS-230). Calibration and quality control were performed according to the manufacturer's instructions and OECD Guideline 425 [19] for acute oral toxicity testing to ensure the reliability of the results.

### 2.8. Necroscopy and Histopathological Examination

Following the treatment period, the rats administered the commonest chemotherapeutic drugs were euthanized via CO_2_ asphyxiation, and necropsy was performed immediately [19]. The liver, kidney, spleen, and stomach were dissected using standard surgical tools: The liver was excised from the abdominal cavity after severing the hepatic ligaments, kidneys were removed post-adrenal detachment, the spleen was isolated from its mesenteric attachments, and the stomach was extracted after esophageal and duodenal transection. Organs were rinsed with ice-cold PBS (pH 7.4) to remove blood and debris and then sectioned into 5-mm-thick samples. The tissue samples were fixed in 10% neutral buffered formalin for 24 h at room temperature, followed by dehydration through a graded ethanol series (70%, 95%, 100%) and clearing in xylene. Samples were embedded in paraffin wax, and 4-μm sections were cut using a rotary microtome (Leica RM2235). Sections were mounted on glass slides, deparaffinized in xylene, and rehydrated in descending ethanol gradients. Staining was performed with hematoxylin (5 min) and eosin (2 min), followed by rinsing in distilled water and dehydration. Slides were coverslipped with dibutylphthalate polystyrene xylene (DPX) mountant. Histopathological analysis was conducted using a light microscope (Olympus BX51) at 100× and 400× magnifications. Five fields per section were examined for morphological changes in hepatocytes, glomeruli/tubules, white/red pulp, and gastric mucosa/submucosa, respectively, after which observations were recorded for samples derived from experimental rats administered PBS (control), cytarabine, cisplatin, doxorubicin, and cyclophosphamide, respectively [21].

### 2.9. Statistical Analysis

Quantitative data from the hematological and biochemical analysis were tabulated on Excel spreadsheets, organized, and then analyzed using GraphPad Prism software (Version 10.3.1, GraphPad Software Inc., San Diego, CA). The results were presented as mean ± standard deviation (SD). Statistical significance was determined using one-way ANOVA followed by Tukey's post hoc test for multiple comparisons, whereby a *p* value of < 0.05 was considered statistically significant. Histopathological data were examined as recommended by OECD Guidelines for the data analysis in toxicity studies [19, 21] and presented.

## 3. Results

### 3.1. Hematological Profile

The hematological analysis demonstrated marked treatment-specific effects across all parameters (*p* < 0.001; Table 1). WBC counts were significantly elevated in rats administered docetaxel and markedly reduced in those given oxaliplatin (both *p* < 0.001; Table 1). Rats treated with doxorubicin, paclitaxel, cisplatin, cytarabine, cyclophosphamide, and controls exhibited intermediate values without significant differences (*p* > 0.001; Table 1). Lymphocyte percentages were the highest in the doxorubicin group, differing significantly from all other treatments except oxaliplatin (*p* < 0.001; Table 1), while docetaxel-treated rats displayed the lowest levels (*p* < 0.001; Table 1). Intermediate percentages were observed in the paclitaxel, cisplatin, cytarabine, cyclophosphamide, and control groups (Table 1).

Neutrophil percentages showed the inverse pattern: significantly increased in the docetaxel group and reduced in the doxorubicin group (both *p* < 0.001; Table 1), with no significant differences among the remaining treatments (*p* > 0.001; Table 1). RBC counts were significantly elevated in cyclophosphamide-treated rats and depressed in those given oxaliplatin (*p* < 0.001; Table 1), while the other groups did not differ significantly (*p* > 0.001; Table 1). Hemoglobin (Hb) concentrations were higher in the doxorubicin and cytarabine groups relative to oxaliplatin (*p* < 0.001; Table 1), whereas docetaxel, paclitaxel, cisplatin, and control groups displayed intermediate levels without significant variation (*p* > 0.001; Table 1).

Platelet counts were significantly elevated in docetaxel-treated rats compared to paclitaxel, cytarabine, and cyclophosphamide groups (*p* < 0.001; Table 1). Cytarabine-treated rats did not differ from controls (*p* > 0.001; Table 1), while the doxorubicin, oxaliplatin, cisplatin, and control groups showed no significant variation (*p* > 0.001; Table 1). MCV was the highest in the oxaliplatin group (*p* < 0.001; Table 1) and significantly lower in cisplatin-treated rats compared with docetaxel, paclitaxel, oxaliplatin, and cyclophosphamide groups (*p* < 0.001; Table 1). Intermediate MCV values were observed in the doxorubicin, cytarabine, and control groups (Table 1). MCH was also significantly elevated following oxaliplatin administration (*p* < 0.001; Table 1), with no significant differences among the remaining groups (*p* > 0.001; Table 1). HCT values were the highest in cytarabine-treated rats (*p* < 0.001; Table 1) and lowest in those administered oxaliplatin and cisplatin (*p* < 0.001; Table 1), whereas docetaxel, doxorubicin, cyclophosphamide, and controls did not differ significantly (*p* > 0.001; Table 1).

### 3.2. Biochemical Markers

The analysis of biochemical markers revealed pronounced organ-specific toxicities, particularly affecting the liver and kidneys (*p* < 0.001; Table 2). Rats administered paclitaxel exhibited significantly elevated aspartate AST and ALP levels compared with all other groups, including controls (*p* < 0.001; Table 2). Cytarabine-treated rats also demonstrated significantly higher ALP and total bilirubin concentrations relative to both the other drug-treated groups and controls (*p* < 0.001; Table 2). Elevated ALB levels were observed in rats administered doxorubicin and cyclophosphamide, which were significantly greater than those recorded in the remaining treatment and control groups (*p* < 0.001; Table 2). Furthermore, paclitaxel- and cyclophosphamide-treated rats displayed significantly increased urea and creatinine levels compared with all other groups, including controls (*p* < 0.001; Table 2).

### 3.3. Concentration of Electrolytes

The electrolyte analysis revealed distinct drug-specific alterations (*p* < 0.001; Table 3). Potassium concentrations were significantly elevated in rats administered oxaliplatin compared with all other treatment groups (*p* < 0.001; Table 3). In contrast, rats treated with doxorubicin and cisplatin exhibited significantly lower potassium levels than the remaining groups (*p* < 0.001; Table 3). Sodium concentrations remained stable across all groups without significant differences (*p* > 0.001; Table 3). Chloride levels were also generally stable, except for cytarabine-treated rats, which demonstrated significantly higher values than all other groups (*p* < 0.001; Table 3).

### 3.4. Histopathological Findings

**Figure 1:**
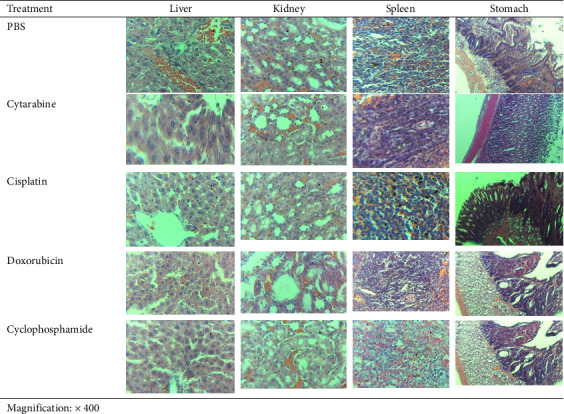
Histological micrographs of various organs isolated from rats administered the commonest anticancer drugs.

Histological examination of liver, kidney, spleen, and stomach tissues from Wistar rats administered cytarabine, cisplatin, doxorubicin, cyclophosphamide, or PBS (control) revealed treatment-specific alterations (Figure 1). In the liver, PBS-administered rats displayed normal architecture characterized by centrally located hepatocyte nuclei, eosinophilic cytoplasm, and open sinusoidal spaces (Figure 1). In contrast, cytarabine- and cyclophosphamide-treated rats showed marked cytoplasmic vacuolation and sinusoidal narrowing, whereas Cisplatin- and doxorubicin-treated groups exhibited milder vacuolation accompanied by occasional perivascular inflammation (Figure 1).

Kidney sections from controls demonstrated intact glomeruli and well-preserved tubular morphology, while cisplatin- and doxorubicin-treated rats showed mild epithelial flattening, reduced brush border height, and luminal debris (Figure 1). Cytarabine- and cyclophosphamide-administered rats exhibited more pronounced changes, including glomerular compaction, narrowing of Bowman's space, and slight interstitial edema (Figure 1).

In the spleen, control samples displayed clearly defined white and red pulp regions. By contrast, doxorubicin- and cyclophosphamide-treated rats showed reduced white pulp cellularity and mild red pulp congestion, whereas cytarabine- and cisplatin-treated groups exhibited subtler reductions accompanied by sporadic hemosiderin deposits (Figure 1).

Examination of stomach tissues revealed intact mucosal structure in controls. Cytarabine- and cisplatin-treated rats demonstrated submucosal lymphocytic infiltration, with cytarabine additionally associated with glandular hyperplasia and focal mucin depletion (Figure 1). Doxorubicin- and cyclophosphamide-administered rats showed milder changes, including epithelial thinning, low-grade inflammation, and increased goblet cell activity (Figure 1).

Notably, across all organs and treatment groups, no evidence of necrosis, hemorrhage, or fibrosis was observed (Figure 1).

## 4. Discussion

Chemotherapeutic agents remain indispensable in cancer treatment, yet their therapeutic benefits are often offset by systemic toxicities that compromise efficacy and patient outcomes [6]. Understanding these organ-specific effects is critical for devising strategies that mitigate adverse reactions and improve the safety of chemotherapy regimens [22]. While most preclinical studies have focused on individual drugs or isolated organ systems [14–16, 23–27], the present study is novel in its comparative evaluation of seven widely used chemotherapeutic agents within a single standardized rat model, simultaneously assessing hematological, biochemical, electrolyte, and histopathological alterations under identical conditions. This integrative design provides a uniquely comprehensive and directly comparable toxicity profile, thereby revealing the heterogeneous and drug-specific nature of chemotherapy-induced toxicities. Importantly, the uniqueness of this study is anchored on its implications for Medication Therapy Management (MTM) in oncology, as cancer care has advanced considerably in treatment options yet still lacks personalized strategies to identify and prevent unforeseen adverse effects associated with chemotherapeutic agents [17, 28, 29]. The absence of a risk management framework that holistically integrates therapeutic, social, and economic outcomes within MTM [28] underscores the novelty and significance of our findings, which contribute to building the evidence base needed to inform such models. The results therefore carry significant implications for clinical practice, supporting the rationale for personalized monitoring protocols and early protective strategies that can optimize therapeutic safety and improve patient outcomes.

The hematological profile revealed significant alterations in WBC, RBC, and platelet counts, reflecting the myelosuppressive and immunomodulatory effects of chemotherapeutic agents [30]. Notably, rats administered docetaxel exhibited marked leukocytosis and thrombocytosis, which may indicate an inflammatory response or bone marrow stimulation [31]. These effects can be linked to docetaxel's mechanism of action, which involves microtubule stabilization leading to mitotic arrest and activation of stress-related signaling pathways [32, 33]. Such cellular stress may induce the release of pro-inflammatory cytokines, including Interleukin 6 (IL-6) and tumor necrosis factor-alpha (TNF-α), thereby promoting leukocyte proliferation and platelet production [32, 34]. Moreover, transient bone marrow stimulation may occur as a compensatory response to peripheral cytotoxic stress, contributing to the observed hematological alterations [35].

In contrast, rats administered doxorubicin or cisplatin exhibited reduced WBC counts and lymphocyte percentages, consistent with immunosuppression [36]. These findings align with clinical evidence showing that anthracyclines and platinum-based agents disrupt hematopoiesis through oxidative damage and apoptosis of bone marrow progenitor cells [37, 38]. The elevated RBC count and hemoglobin levels in cyclophosphamide-treated rats suggest minimal suppression of erythropoiesis, contrasting with the anemia observed in oxaliplatin- and cisplatin-treated groups [39, 40]. This variability in hematological toxicity highlights the importance of drug-specific mechanisms, treatment regimens, and individual susceptibility, reinforcing the need for regular monitoring and supportive interventions such as growth factor therapy or transfusion to manage chemotherapy-induced cytopenias [40].

Biochemical markers are indispensable tools for assessing drug toxicity and safety evaluation [41]. In the present study, rats administered various anticancer drugs exhibited significant hepatotoxic and nephrotoxic alterations, consistent with the known organ-specific toxicity of chemotherapeutic agents [42]. Paclitaxel-treated rats showed significantly elevated aspartate AST and ALP levels, indicative of hepatocellular injury [41, 43]. Similarly, cytarabine administration resulted in markedly increased ALP and total bilirubin levels, underscoring its hepatotoxic potential [42, 43]. These findings align with previous reports describing drug-induced liver injury (DILI) as a common adverse effect of chemotherapy, particularly for agents metabolized in the liver [42].

Interestingly, in this study, cisplatin administration did not significantly alter hepatological parameters such as AST, ALP, or bilirubin, despite its well-documented hepatotoxic potential in the literature [42, 43]. This discrepancy may reflect dose-dependent or duration-dependent effects. Previous studies demonstrating cisplatin-induced hepatotoxicity often employed higher doses or prolonged exposure periods, leading to greater oxidative stress and hepatic injury [44, 45]. Additionally, interspecies and strain differences in metabolism and susceptibility to oxidative damage may contribute to the observed variations [46–48]. Therefore, the relatively mild hepatic response observed in the present study may be attributable to the cisplatin dose and exposure duration used, which may have been sufficient to induce renal toxicity but not overt hepatotoxicity.

The elevated urea and creatinine levels in cisplatin- and cytarabine-treated rats are consistent with their established nephrotoxic profiles, primarily mediated through tubular damage and oxidative stress [42, 49]. Elevated albumin levels in doxorubicin- and cyclophosphamide-treated rats may reflect compensatory mechanisms or altered protein metabolism [39], warranting further investigation. Collectively, these findings highlight the importance of considering dose, treatment duration, and species-specific factors when interpreting chemotherapy-induced organ toxicity and reinforce the need for routine monitoring of hepatic and renal function in patients receiving cytotoxic agents.

Electrolyte imbalances are important indicators of chemotherapy-induced systemic toxicity [50]. In this study, oxaliplatin caused hypokalemia, while doxorubicin and cisplatin similarly induced potassium depletion in experimental rats. These alterations can be attributed to drug-specific effects on renal tubular function and cellular ion homeostasis [51]. Cisplatin is known to impair potassium reabsorption in the distal convoluted tubule and collecting duct by damaging tubular epithelial cells through oxidative stress, mitochondrial dysfunction, and inhibition of Na^+^/K^+^-ATPase activity [52–54]. Doxorubicin-induced hypokalemia may arise from cardiotoxicity-associated catecholamine release and increased renal potassium excretion secondary to tubular oxidative injury [55]. Besides, oxaliplatin's metabolism can generate reactive oxygen species (ROS) and modulate calcium-dependent potassium channels, leading to intracellular potassium shifts and renal loss [56, 57].

The relatively stable sodium and chloride levels across most treatment groups suggest that glomerular filtration and proximal tubular sodium-chloride cotransport were largely preserved despite histological evidence of renal injury. This preservation may be due to the compensatory activation of the renin–angiotensin–aldosterone system (RAAS), which enhances sodium reabsorption and maintains extracellular volume homeostasis under nephrotoxic stress [58]. The early emergence of hypokalemia, preceding marked azotemia, indicates that electrolyte imbalance may precede overt acute kidney injury (AKI) and, thus, represents a sensitive biomarker of renal tubular dysfunction [49, 50].

These findings underscore the clinical importance of routine electrolyte monitoring during chemotherapy and highlight the potential utility of renoprotective agents such as amifostine, which scavenges free radicals and preserves tubular integrity [59–61]. Future research should further elucidate the molecular pathways underlying these systemic effects, with an emphasis on oxidative stress modulation, mitochondrial protection, and transporter regulation. The development of targeted therapies that minimize off-target effects on renal ion channels, hematopoiesis, and hepatic metabolism could significantly improve treatment safety. Moreover, integrating novel biomarkers, such as microRNAs for hepatotoxicity and neutrophil gelatinase-associated lipocalin (NGAL) for nephrotoxicity [62], may enable early detection and timely intervention, thereby reducing the burden of chemotherapy-related complications.

Chemotherapeutic agents, while indispensable in the treatment of cancer, are often associated with significant organ-specific toxicity, which can limit their therapeutic efficacy and compromise patient outcomes [9, 39]. Understanding the histopathological effects of these agents is crucial for developing strategies to mitigate their adverse effects and improve the safety profile of chemotherapy regimens. The liver's response to anticancer drug administration in this study, characterized by extensive cytoplasmic vacuolation and sinusoidal narrowing in cytarabine- and cyclophosphamide-administered rats, aligns with previous findings of chemotherapeutic-induced hepatotoxicity [39, 63]. However, the absence of necrosis or fibrosis here suggests that the current regimen induces an early, reversible stress response, possibly linked to oxidative damage or impaired lipid metabolism [64]. The presence of perivascular inflammation in cisplatin-administered group and doxorubicin-administered group further hints at an immune-mediated component, a less-explored aspect of drug hepatotoxicity [65, 66]. Although these chemotherapeutic agents were associated with systemic immunosuppression, localized hepatic inflammation may still occur due to direct cellular injury or ROS-mediated endothelial activation [65]. This local response could involve the recruitment of resident macrophages (Kupffer cells) and neutrophils to the perivascular regions as part of an early inflammatory defense, even in the context of overall leukopenia. Such concurrent systemic immunosuppression and local inflammation are not contradictory but rather reflect distinct tissue-level responses to cytotoxic stress [65, 66]. These observations reinforce the complex interplay between immune modulation and organ-specific toxicity in chemotherapy and highlight the potential for inflammatory mediators to contribute to hepatic injury even when systemic immunity is compromised.

In the kidney, mild tubular epithelial flattening, reduced brush borders, and glomerular compaction were observed in cisplatin- and doxorubicin-administered rats, reflecting early nephrotoxic changes commonly associated with these agents [67, 68]. Cisplatin's nephrotoxicity is mechanistically linked to its accumulation in proximal tubular cells via organic cation transporters, where it induces mitochondrial dysfunction, oxidative stress, and apoptosis through activation of the p53 and MAPK pathways [69, 70]. Doxorubicin, on the other hand, generates ROS via the redox cycling of its quinone moiety, leading to lipid peroxidation and mitochondrial injury in renal tissues [71]. The absence of overt tubular necrosis or casts differentiates this model from severe AKI, suggesting that the observed histological changes represent an early or subclinical phase of toxicity [72]. The interstitial edema noted in cytarabine- and cyclophosphamide-administered rats indicates disruption of renal fluid dynamics, potentially mediated by oxidative stress and inflammatory cytokine release during drug metabolism and excretion [73]. This observation, which is not widely emphasized in the previous literature, may reflect altered renal microcirculation and endothelial permeability due to free radical accumulation.

The spleen's reduced white pulp cellularity and red pulp congestion in treated samples, particularly in doxorubicin- and cyclophosphamide-administered rats, deviate from the broad immunosuppression typically reported with cytotoxic therapy [74, 75]. Doxorubicin's intercalation into DNA and inhibition of Topoisomerase II induce apoptosis in proliferating lymphoid cells, explaining the reduced splenic cellularity [76]. Cyclophosphamide exerts selective lymphoid toxicity through the formation of its active metabolite phosphoramide mustard, which cross-links DNA in rapidly dividing lymphocytes [77]. The observed hemosiderin deposits in cytarabine- and cisplatin-treated groups suggest increased erythrocyte turnover or mild hemolysis, consistent with subclinical oxidative injury to RBCs [78]. However, the absence of splenic atrophy seen in higher-dose regimens [79, 80] implies a dose-dependent or selective lymphoid impact rather than systemic cytotoxicity. Collectively, these findings indicate that the spleen's histological alterations may represent a balance between drug-induced oxidative stress and compensatory reticuloendothelial activation.

The stomach's submucosal inflammation and glandular hyperplasia in cytarabine and cisplatin-administered rats [81, 82] contrast with the ulceration often seen in methotrexate-based studies, suggesting a milder, possibly adaptive response to drug exposure [80, 83]. Cytarabine's incorporation into gastric epithelial DNA can trigger low-grade apoptotic signaling, stimulating regenerative hyperplasia, while cisplatin may activate NF-κB-mediated inflammatory cascades, leading to submucosal infiltration [84]. The epithelial thinning and goblet cell increase in the doxorubicin-administered group and cyclophosphamide-administered group suggest a protective mucin adaptation, whereby mucosal surfaces enhance mucin secretion in response to oxidative and inflammatory stimuli, which is a novel finding compared to prior focus on mucosal erosion [83]. This mucosal remodeling likely reflects an early defense mechanism aimed at preserving barrier integrity under chemotherapeutic stress. This implies that gastric responses may serve as an early indicator of gastrointestinal tolerance, prompting research into mucin dynamics and inflammatory mediators. Clinically, these subtle changes support the use of prophylactic gastroprotection in patients to mitigate progression to overt damage, enhancing treatment adherence [85].

In the context of the existing literature, the heterogeneity and agent-specificity observed in this study underscore the importance of personalized toxicity monitoring. Unlike single-agent studies, our comparative design strengthens the translational impact by allowing clinicians to weigh trade-offs between therapeutic efficacy and anticipated toxicities across drug classes. For example, nephrotoxicity with cisplatin and hepatotoxicity with cytarabine observed here mirror adverse events reported in oncology patients, reinforcing the translational relevance of our findings. By integrating hematological, biochemical, electrolyte, and histopathological assessments, this study provides a multidimensional analysis that can inform more holistic monitoring frameworks in clinical oncology. However, we acknowledge that the precise molecular or cellular mechanisms underlying the observed toxicities were not elucidated. While this limits mechanistic novelty, our results establish a robust comparative baseline that future studies can build upon to identify the pathways mediating these toxicities.

### 4.1. Study Limitations

While this study provides valuable insights into the systemic effects of chemotherapeutic agents, it has several limitations. First, although it demonstrates drug-specific systemic toxicity patterns, the precise mechanisms of action responsible for these toxicities were not determined. Identifying molecular mediators (e.g., oxidative stress, apoptosis pathways, or inflammatory cascades) would add mechanistic novelty and deepen translational insights and should be addressed in follow-up studies. Second, the use of a single dose for each agent may not fully capture dose-dependent toxicity, limiting extrapolation across therapeutic ranges. Third, the 14-day observation period, although compliant with OECD Guideline 425, may not reflect long-term or cumulative effects; chronic toxicity studies with extended follow-up are warranted. Finally, while the rodent model offers valuable preclinical insights, interspecies differences necessitate cautious extrapolation to human oncology practice.

## Figures and Tables

**Table 1:** Hematological profile of rats administered various anticancer drugs.

Treatment group	WBC (× 10^3^/UL)	Lymphocyte (%)	Neutrophil (%)	RBC (× 10^6^/UL)	Hb (g/dL)	Platelet (× 10^3^/UL)	MCV (%)	MCH (Pg)	HCT (g/dL)
Group 1	27.80 ± 9.84^a^	9.18 ± 0.59^c^	30.02 ± 21.83^a^	5.72 ± 0.27^ab^	14.02 ± 0.72^ab^	611.80 ± 58.59^a^	72.38 ± 1.61^b^	24.54 ± 0.09^b^	41.26 ± 0.91^a^
Group 2	9.30 ± 2.20^b^	87.36 ± 2.64^a^	6.66 ± 2.05^b^	6.44 ± 0.09^ab^	15.3 ± 0.21^a^	467.20 ± 34.51^ab^	65.94 ± 0.74^cd^	23.8 ± 0.35^b^	42.23 ± 1.00^a^
Group 3	13.04 ± 1.92^b^	79.08 ± 2.86^ab^	11.98 ± 1.39^b^	5.86 ± 0.09^ab^	14.56 ± 0.31^ab^	433.60 ± 12.07^b^	70.18 ± 0.27^bc^	24.84 ± 0.13^b^	41.34 ± 0.87^a^
Group 4	7.14 ± 2.68^b^	84.34 ± 1.59^ab^	8.78 ± 0.18^b^	4.14 ± 0.59^b^	11.40 ± 2.14^b^	461.60 ± 149.44^ab^	86.68 ± 5.77^a^	27.64 ± 3.60^a^	35.67 ± 4.68^b^
Group 5	8.30 ± 1.70^b^	77.20 ± 11.02^ab^	13.20 ± 6.54^b^	5.64 ± 0.54^ab^	13.38 ± 1.39^ab^	481.00 ± 92.76^ab^	63.52 ± 1.07^d^	23.68 ± 0.67^b^	35.93 ± 3.50^b^
Group 6	9.24 ± 1.23^b^	81.74 ± 2.13^ab^	11.54 ± 2.17^b^	6.52 ± 0.28^ab^	15.94 ± 0.80^a^	414.60 ± 53.14^b^	66.66 ± 1.08^cd^	24.42 ± 0.27^b^	43.41 ± 1.52^a^
Group 7	10.22 ± 1.83^b^	74.48 ± 6.01^b^	16.68 ± 4.90^b^	8.06 ± 3.72^a^	15.62 ± 0.68^a^	431.00 ± 81.61^b^	68.96 ± 4.50^bc^	24.78 ± 0.51^b^	43.29 ± 2.29^a^
Group 8	10.84 ± 4.00^b^	80.86 ± 13.10^ab^	7.85 ± 5.38^b^	6.16 ± 0.79^ab^	12.98 ± 3.93^ab^	526.40 ± 139.52^ab^	68.02 ± 2.33^bcd^	24.18 ± 0.50^b^	41.83 ± 4.37^a^

*Note:* Values are presented as $\bar{x}\pm \text{SD},n=5.$ Values with different superscript alphabets within the same column are significantly different (*p* < 0.001; one-way ANOVA with Tukey's post hoc). Group 1: Docetaxel (10 mg/kg bw); Group 2: Doxorubicin (10 mg/kg bw); Group 3: Paclitaxel (4 mg/kg bw); Group 4: Oxaliplatin (4 mg/kg bw); Group 5: Cisplatin (2 mg/kg bw); Group 6: Cytarabine (50 mg/kg bw); Group 7: Cyclophosphamide (10 mg/kg bw); Group 8: Phosphate-buffered saline (10 mL/kg bw).

**Table 2:** Biochemical markers in rats administered various anticancer drugs.

Treatment group	AST (U/L)	ALP (U/L)	GGT (U/L)	ALB (g/dL)	TP (g/dL)	Direct bilirubin (mg/dL)	Total bilirubin (mg/dL)	Urea (mg/L)	Creatinine (mmol/L)
Group 1	59.00 ± 11.31^b^	102.00 ± 21.21^c^	69.00 ± 2.83^ab^	10.00 ± 1.41^b^	0.23 ± 0.02^d^	0.35 ± 0.07^c^	4.10 ± 0.28^b^	47.08 ± 2.42^c^	0.58 ± 0.19^ab^
Group 2	48.40 ± 6.84^bc^	205.60 ± 95.62^bc^	62.80 ± 9.81^b^	71.40 ± 11.55^a^	0.27 ± 0.09^d^	2.02 ± 0.58^b^	3.56 ± 0.47^b^	53.42 ± 3.86^abc^	0.81 ± 0.23^a^
Group 3	141.00 ± 14.72^a^	304.75 ± 10.97^a^	82.25 ± 2.99^a^	10.00 ± 1.41^b^	0.59 ± 0.52^d^	0.89 ± 0.17^c^	3.12 ± 0.34^b^	62.21 ± 3.84^a^	0.49 ± 0.05^b^
Group 4	40.00 ± 1.00^cd^	250.00 ± 71.46^ab^	64.00 ± 2.65^b^	8.33 ± 1.53^b^	2.29 ± 1.72^bc^	3.65 ± 1.14^a^	4.10 ± 1.59^b^	53.52 ± 4.52^abc^	0.44 ± 0.06^b^
Group 5	48.25 ± 2.22^bc^	296.00 ± 6.98^ab^	51.00 ± 1.63^c^	15.50 ± 2.89^b^	3.35 ± 0.42^b^	2.85 ± 0.17^ab^	6.26 ± 0.34^a^	55.68 ± 14.42^abc^	0.76 ± 0.12^a^
Group 6	53.40 ± 2.70^bc^	298.80 ± 7.89^a^	33.20 ± 3.96^d^	12.80 ± 3.03^b^	5.21 ± 0.75^a^	3.43 ± 0.17^a^	6.33 ± 0.52^a^	53.60 ± 7.95^abc^	0.50 ± 0.04^b^
Group 7	47.00 ± 5.83^bc^	304.40 ± 4.78^a^	57.20 ± 6.34^bc^	72.00 ± 10.91^a^	0.35 ± 0.38^d^	0.45 ± 0.19^c^	2.94 ± 0.32^b^	60.05 ± 6.50^abc^	0.47 ± 0.12^b^
Group 8	27.00 ± 2.16^d^	127.75 ± 35.26^c^	11.50 ± 3.11^e^	9.50 ± 1.29^b^	1.20 ± 0.37^cd^	0.73 ± 0.13^c^	4.05 ± 0.34^b^	48.43 ± 4.90^bc^	0.61 ± 0.05^ab^

*Note:* Values are presented as $\bar{x}\pm \text{SD},n=5.$ Values with different superscript alphabets within the same column are significantly different (*p* < 0.001; one-way ANOVA with Tukey's post hoc). Group 1: Docetaxel (10 mg/kg bw); Group 2: Doxorubicin (10 mg/kg bw); Group 3: Paclitaxel (4 mg/kg bw); Group 4: Oxaliplatin (4 mg/kg bw); Group 5: Cisplatin (2 mg/kg bw); Group 6: Cytarabine (50 mg/kg bw); Group 7: Cyclophosphamide (10 mg/kg bw); Group 8: Phosphate-buffered saline (10 mL/kg bw).

**Table 3:** Concentration of electrolytes in rats administered various anticancer drugs.

Treatment group	Sodium (mmol/L)	Chloride (mmol/L)	Potassium (mmol/L)
Group 1	146.12 ± 4.34^a^	106.15 ± 1.36^ab^	4.83 ± 0.77^ab^
Group 2	146.95 ± 6.02^a^	99.89 ± 3.32^b^	3.80 ± 0.33^c^
Group 3	147.37 ± 8.48^a^	105.42 ± 6.69^ab^	4.40 ± 0.68^abc^
Group 4	150.70 ± 6.74^a^	103.73 ± 6.22^ab^	5.30 ± 0.88^a^
Group 5	150.16 ± 9.20^a^	106.85 ± 3.76^ab^	3.75 ± 0.26^c^
Group 6	147.13 ± 8.83^a^	112.37 ± 12.41^a^	4.51 ± 0.38^abc^
Group 7	144.43 ± 15.35^a^	105.06 ± 8.48^ab^	4.29 ± 0.13^bc^
Group 8	141.54 ± 2.12^a^	102.57 ± 2.56^ab^	3.63 ± 0.03^c^

*Note:* Values are presented as $\bar{x}\pm \text{SD},n=5.$ Values with different superscript alphabets within the same column are significantly different (*p* < 0.001; one-way ANOVA with Tukey's post hoc). Group 1: Docetaxel (10 mg/kg bw); Group 2: Doxorubicin (10 mg/kg bw); Group 3: Paclitaxel (4 mg/kg bw); Group 4: Oxaliplatin (4 mg/kg bw); Group 5: Cisplatin (2 mg/kg bw); Group 6: Cytarabine (50 mg/kg bw); Group 7: Cyclophosphamide (10 mg/kg bw); Group 8: Phosphate-buffered saline (10 mL/kg bw).

## Data Availability

All data are in the manuscript and/or supporting information files, and any additional information may be provided by the corresponding author upon reasonable request.
